# 90-degree incision in Mohs micrographic surgery for eyelid margin tumors – Is there a benefit?^☆^

**DOI:** 10.1016/j.abd.2023.06.002

**Published:** 2023-09-22

**Authors:** Glaysson Tassara Tavares, Isabela Boechat Morato, Alberto Julius Alves Wainstein

**Affiliations:** a Service of Dermatology, Hospital das Clínicas, Universidade Federal de Minas Gerais, Belo Horizonte, MG, Brazil; b Faculdade de Ciências Médicas de Minas Gerais, Belo Horizonte, MG, Brazil

Dear Editor,

Although excision with a 45-degree angled scalpel (A45) is the reference technique in Mohs Micrographic Surgery (MMS),^1^ it can pose difficulties in tumors located at the eyelid margin (TUEM),^2^ on the other hand, excision without angulation (with scalpes at 90 degrees) has been described for this location.^3^

The aim of this study was to assess whether resection at a 90-degree angle (A90) for TUEM had the same efficacy as the classic excision. This is a cross-sectional retrospective study analyzing the medical records of patients with keratinocyte carcinomas, treated by the same Mohs surgeon and approved by the ethics committee of the institution under CAAE n. 17621419.3.0000.5134. The following data were collected: age, sex, primary or recurrent tumor, diameter, histopathological type, number of phases in MMS, scalpel angle at 45° or 90° and recurrence rate. The patients were followed for healing control.

Patients seen between 2008 and April 2019, with tumors located exclusively on the eyelid margin, who underwent MMS were included. Patients with tumors other than keratinocytic carcinoma were excluded. MMS using the A45^4^ technique was performed in patients up to the year 2013 and with the A90 technique from 2014 onwards, thus forming the two groups that were evaluated.

The first group was treated with the classic technique, with tumor debulking and A45 scalpel margin excision.^1^ In the second group, *en bloc* excision of the tumor with a 2-mm margin was performed (Fig. 1), with an A90 scalpel. After the excision, debulking was performed *ex vivo* (Fig. 2). The resulting margin was usually included as a single fragment (Figure 3, Figure 4).

**Figure 1:**
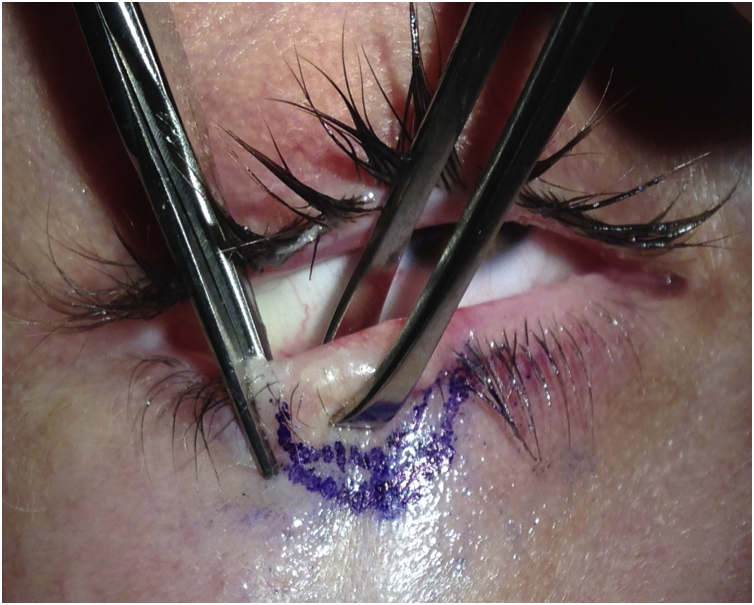
90-degree excision

**Figure 2:**
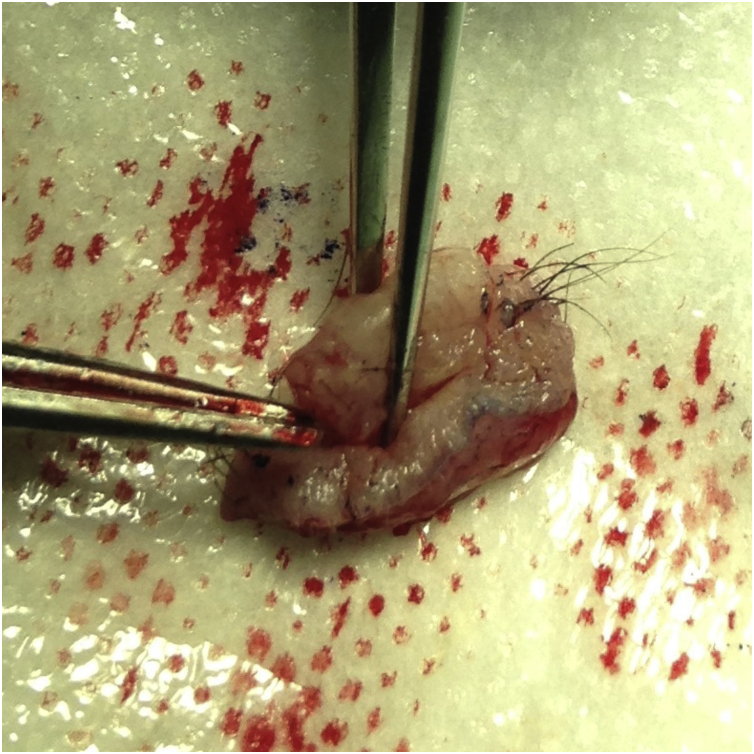
*Ex vivo* debulking

**Figure 3:**
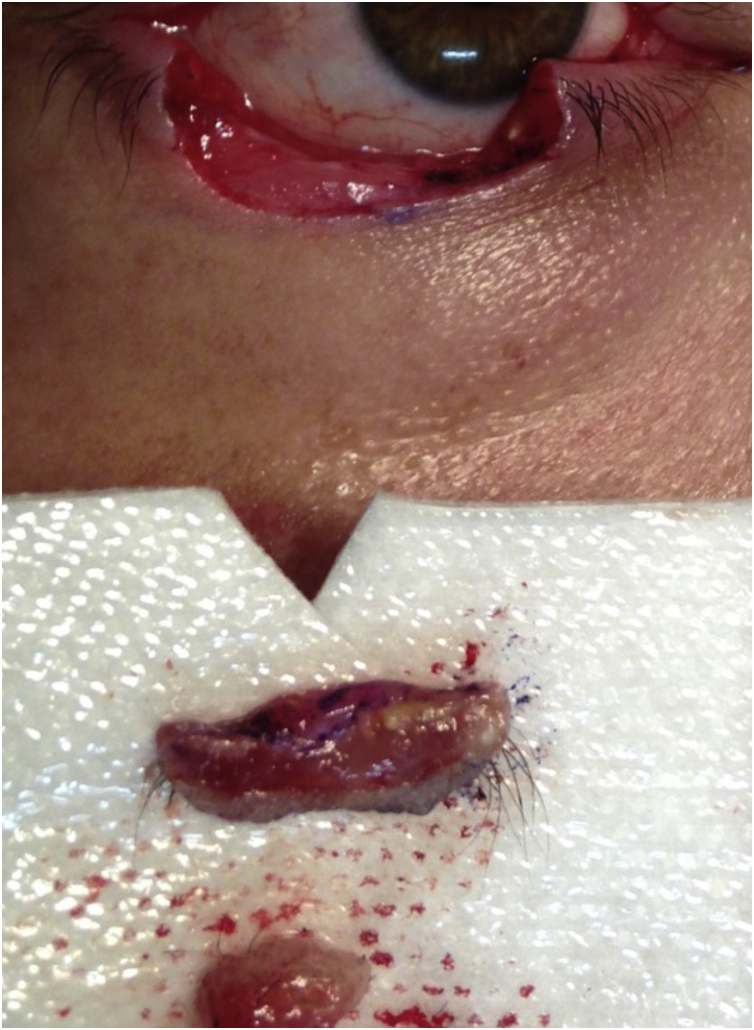
Lateral/deep margin in contact with Telfa

**Figure 4:**
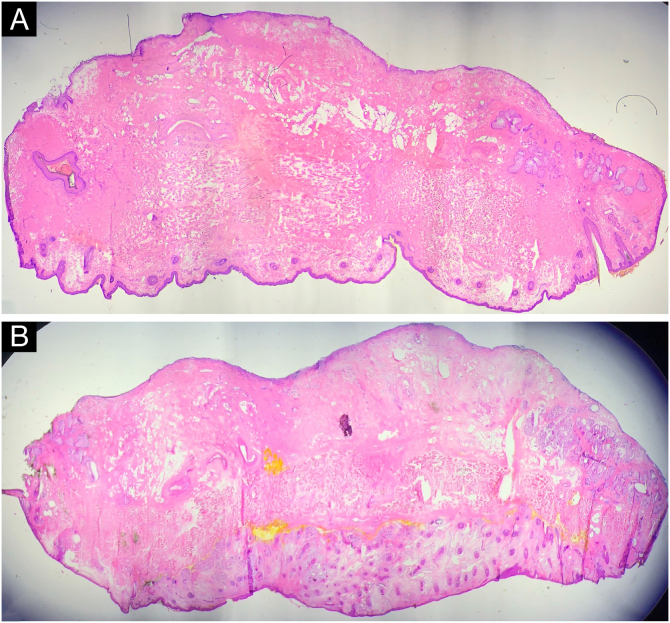
Histopathology of two cases with 2.5 magnification stained with Hematoxylin & eosin showing the entire lateral and deep margin

The inferential analysis was performed with Fisher's Exact Test (R software) and p < 0.05 was considered significant.

A total of 59 patients were identified with tumors on the eyelid margin, with a mean age of 68.3 ± 16.45 years; 57.6% were women and 79% had basal cell carcinoma (BCC). The A45 excision was performed in 35 (57.9%) patients and A90 in 24 patients (42.1% of cases). As for risk factors; 52.6% had a high-risk histopathology type; 47.4% had a diameter >10 mm; 96.4% of the tumors were primary ones. The average number of stages for MMS was 2.33 ± 1.16.

In both groups (A45 and A90), the analyzed risk factors were similar: high-risk histopathological type (p = 0.11), diameter >10 mm (p = 0.593; Table 1). The number of stages required during surgery was similar (p = 0.215; Table 2). There were two (3.57%) recurrences in the A45 group and none in the A90 group (p = 0.504). The mean follow-up time was 64.8 months.

**Table 1:** Variables: incision angle, histopathological risk evaluation and tumor diameter

	Histopathological risk evaluation		Diameter	
	High (p = 0.11)	Low	≤ 10 mm (p = 0.593)	> 10 mm
**45º**	14 (43.8%)	18 (56.2%)	17 (51.5%)	16 (48.5%)
**90º**	16 (66.7%)	8 (33.3%)	10 (41.7%)	14 (58.3%)
**Total**	30 (53.6%)	26 (46.4%)	27 (47.4%)	30 (52.6%)

**Table 2:** Variables: angle, number of stages, and recurrence rate

	N. of stages (p = 0.215)		Recurrence (p = 0.504)		Total
	Mean	Median	Yes	No	
**45º**	2.5 (SD 1.2)	2 (min. 1; max. 5)	2 (6.2%)	30 (93.8%)	32 (58.2%)
**90º**	2.1 (SD 1.1)	2 (min. 1; max. 4)	0 (0%)	23 (100%)	24 (42.1%)

Clark's study of eyelid reconstruction after MMS showed that 80% of the cases were BCC, with a mean age of 65 years; 68% were female.^5^

In classic MMS technique (A45), after debulking the TUEM, it is difficult to observe and completely excise the lateral and deep margins, since the exposed dermis and orbicular muscle resemble the conjunctiva. Also, there is loss of tension in the eyelid margin wound, making it difficult to excise these tissues of different consistencies in the same plane. These factors may compromise margin control in MMS.

On the other hand, a 90-degree *en bloc* margin and tumor excision can facilitate full-thickness excision and processing of the eyelid (anterior and posterior lamella). The rigidity of the tarsus (in this excised fragment) allows tumor debulking to be performed *ex vivo*. The tumor observation (obtained from debulking) under microscopy is important, as it is used as a parameter for checking the margin. In the technique described as ‘open book’, debulking is not performed.^2^

According to Karen, the perpendicular excision (A90) and the special way of processing the specimen obtained from TUEMs are important in MMS. This is due to multiple planes with varying tissue consistency.^6^ In their article, three techniques are described for processing the specimen removed perpendicularly in MMS. However, there is no mention in the literature and no statistical analysis of the effectiveness of these techniques.

In Buffo's study, the A90-degree MMS variation showed to be efficient for the treatment of rare skin tumors (non-TUEM), with separation of lateral and deep specimens,^7^ which is not necessary for eyelid tumors.

In the present study, comparison of tumor recurrence rate between excision at A45 *versus* A90 showed no statistical difference between the two groups (p = 0.504). The general recurrence rate was 3.6%, corroborating data in the literature, which ranges from 1.3% to 5.9%.^8^, ^9^, ^10^

According to Mori, for surgical defects in which a portion of the tarsus is resected, the precise alignment of all its structures (lamellae) is crucial to maintain eyelid support and function, avoiding complications.^2^ The A90-degree excision facilitates this reconstruction, which does not occur in the A45 tecnique, requiring complementation and excision of normal tissue, after obtaining a free margin.

As the results of relapse for excision at 45 ° and 90 ° in this study were comparable, excision at 90 ° in MMS may be an option for TUEMs, facilitating excision, specimen processing, and reconstruction. Studies with a larger sample will be relevant to evaluate the technique.

## Financial support

None declared.

## Authors' contributions

Glaysson Tassara Tavares: Design and planning of the study; data collection; drafting and editing of the manuscript; critical review of intellectual content; approval of the final version of the manuscript.

Isabela Boechat Morato: Drafting and editing of the manuscript and critical review of relevant intellectual content; critical review of the intellectual content; approval of the final version of the manuscript.

Alberto Julius Alves Wainstein: Design and planning of the study; data collection; critical review of intellectual content; approval of the final version of the manuscript.

## Conflicts of interest

None declared.
